# Limonoids from the Seeds of *Swietenia macrophylla* and Their Anti-Inflammatory Activities

**DOI:** 10.3390/molecules201018551

**Published:** 2015-10-12

**Authors:** Li-Chai Chen, Hsiang-Ruei Liao, Pei-Yu Chen, Wen-Lung Kuo, Tsung-Hsien Chang, Ping-Jyun Sung, Zhi-Hong Wen, Jih-Jung Chen

**Affiliations:** 1 Department of Marine Biotechnology and Resources, Asia-Pacific Ocean Research Center, National Sun Yat-Sen University, Kaohsiung 80424, Taiwan; E-Mails: icupdrab@gmail.com (L.-C.C.); wzh@mail.nsysu.edu.tw (Z.-H.W.); 2 Department of Pharmacy, Zuoying Branch of Kaohsiung Armed Forces General Hospital, Kaohsiung 81342, Taiwan; E-Mail: jjc5711@yahoo.com.tw; 3 Graduate Institute of Natural Products, School of Traditional Chinese Medicine, College of Medicine, Chang Gung University, Taoyuan 33302, Taiwan; E-Mail: liaoch@mail.cgu.edu.tw; 4 Chung-Jen Junior College of Nursing, Health Sciences and Management, Chiayi 60077, Taiwan; E-Mail: m049@cjc.edu.tw; 5 Department of Medical Education and Research, Kaohsiung Veterans General Hospital, Kaohsiung 813, Taiwan; E-Mail: changth@vghks.gv.tw; 6 National Museum of Marine Biology and Aquarium, Pingtung 944, Taiwan; E-Mail: pjsung@nmmba.gov.tw; 7 Department of Pharmacy, Tajen University, Pingtung 90741, Taiwan; 8 Graduate Institute of Pharmaceutical Technology, Tajen University, Pingtung 90741, Taiwan

**Keywords:** *Swietenia macrophylla*, Meliaceae, structure elucidation, limonoid, anti-inflammatory activity

## Abstract

A new limonoid, swietemacrophin (**1**), was isolated from the seeds of *Swietenia macrophylla*, together with five known compounds **2**–**6**. The structure of **1** was determined through extensive 1D/2D-NMR and mass-spectrometric analyses. Swietemacrophin (**1**), humilinolide F (**2**), 3,6-*O*,*O*-diacetylswietenolide (**3**), 3-*O*-tigloylswietenolide (**4**), and swietemahonin E (**5**) exhibited inhibition (IC_50_ values ≤ 45.44 μM) of superoxide anion generation by human neutrophils in response to formyl-L-methionyl-L-leucyl-L-phenylalanine (fMLP). Compounds **1**, **4**, **5**, and swietenine (**6**) showed potent inhibition with IC_50_ values ≤ 36.32 μM, against lipopolysaccharide (LPS)-induced nitric oxide (NO) generation.

## 1. Introduction

*Swietenia macrophylla* King (Meliaceae) is a tropical timber tree, natively distributed throughout tropical regions of the Americas, mainly in Mexico, Bolivia and Central America. Limonoids [1,2,3,4,5,6], steroids [6], and their derivatives are widely distributed in plants of the genus *Swietenia*. Many of these limonoid derivatives exhibit anti-inflammatory [6], antimalarial [7], and antifungal [8] activities. Limonoids are derived from tetracyclic triterpenes similar to euphol (H-20β) or tirucallol (H-20α) by a series of oxidative changes, interspersed with molecular rearrangements.

Reactive oxygen species (ROS) [e.g., superoxide anion (O_2_^•−^), hydrogen peroxide] and granule proteases (e.g., elastase, cathepsin G) produced by human neutrophils contribute to the pathogenesis of inflammatory diseases. Nitric oxide (NO) is a mediator in the inflammatory response involved in host defense [9]. Suppression of the extensive or inappropriate activation of neutrophils and/or macrophages by drugs has been proposed as a way to ameliorate inflammatory diseases. The effects on pro-inflammatory responses of isolates were evaluated by suppressing fMLP-induced O_2_^•−^ generation by human neutrophils and by inhibiting LPS-induced NO release by murine macrophages.

**Figure 1:**
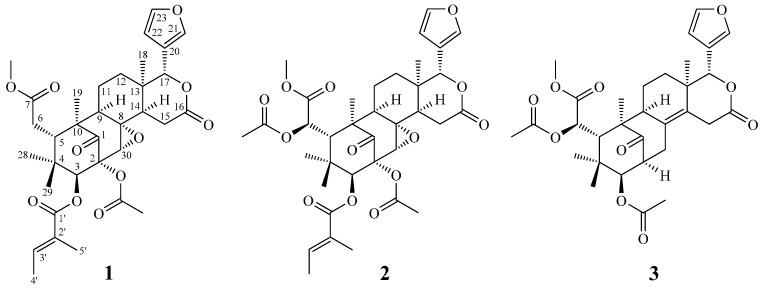

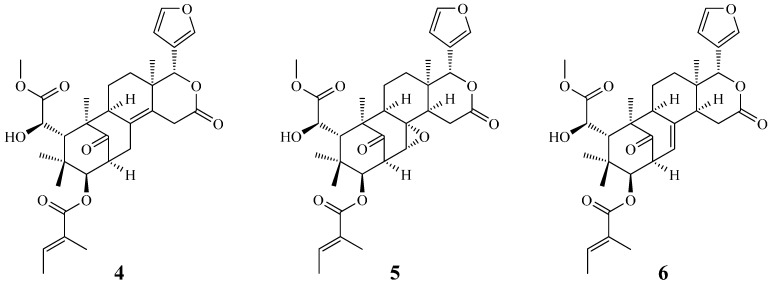
The chemical structures of new compound **1** and known compounds **2**–**6** isolated from *S. macrophylla*.

In a screening program searching for anti-inflammatory compounds from Formosan plants [10,11,12,13,14], *S. macrophylla* has been found to be an active species. The MeOH extract of the seed of *S. macrophylla* showed potent inhibitory effects on superoxide anion generation by human neutrophils in response to fMLP and on NO generation by murine macrophages in response to LPS. The structures of new compound, swietemacrophin (**1**) and five known compounds **2**–**6** have been isolated from the seed of *S. macrophylla* and identified and their structures are depicted in Figure 1. This paper describes the structural elucidation of the compound **1**, and the anti-inflammatory activities of all isolates.

## 2. Results and Discussion

### 2.1. General

Chromatographic purification of the EtOAc-soluble fraction of a MeOH extract of the seeds of *S. macrophylla* on a silica gel column and preparative thin-layer chromatography (TLC) afforded the new compound **1** and five known compounds **2**–**6**.

### 2.2. Structure Elucidation of the New Limonoid

Swietemacrophin (**1**) was isolated as colorless needles. Its molecular formula, C_34_H_42_O_11_, was determined on the basis of the positive HR-ESI-MS at *m/z* 649.2622 [M + Na]^+^ (calcd 649.2625) and was supported by the ^1^H-, ^13^C-, and DEPT NMR data. The presence of carbonyl groups was revealed by the bands at 1735, 1721, and 1708 cm^−1^ in the IR spectrum, and was confirmed by the resonances at δ 166.8, 169.3, 171.0, 174.2, and 206.1 in the ^13^C-NMR spectrum. The ^1^H-NMR spectrum (Table 1) indicated the presence of a methoxy group [δ_H_ 3.74 (3H, s, OMe-7)], an acetoxy group [δ_H_ 2.18 (3H, s, OAc-2)], four tertiary methyl groups [δ_H_ 0.79 (3H, s, Me-28), 0.81 (3H, s, Me-29), 1.01 (3H, s, Me-18), 1.17 (3H, s, Me-19)], a tigloyloxy moiety [δ_H_ 1.93 (3H, br. d, *J* = 7.0 Hz, Me-4′), 1.97 (3H, br s, Me-5′), 7.04 (1H, br q, *J* = 7.0 Hz, H-3′)], three proton resonances of a β-substituted furan ring [δ_H_ 6.44 (1H, br. s, H-22), 7.43 (1H, br. s, H-23), 7.49 (1H, br. s, H-21)], and three oxygenated methines [δ_H_ 3.49 (1H, s, H-30), 5.18 (1H, s, H-17), 5.71 (1H, s, H-3)]. Comparison of the ^1^H- and ^13^C-NMR data (Table 1 and Table 2) of **1** with those of humilinolide F (**2**) [15] suggested that their structures were closely related, except that H-6 [δ_H_ 2.33–2.37 (1H, m)] of **1** replaced the OAc-6 group of humilinolide F (**2**) [15]. This was supported by both HMBC correlations (Table 1) between H-6 (δ_H_ 2.33–2.37) and C-4 (δ_C_ 40.2), C-5 (δ_C_ 42.4), C-7 (δ_C_ 174.2), and C-10 (δ_C_ 50.5), and NOESY correlations between H-6 (δ_H_ 2.33–2.37) and both Me-19 (δ_H_ 1.17) and Me-28 (δ_H_ 0.79). The NOESY cross-peaks (Table 1) of H-3/OAc-2, H-5/Me-29, H-6/H-19, H-6/Me-28, Me-18/H-14, Me-18/H-21, Me-19/H-9, and H-3′/Me-29 evidenced that OAc-2, MeOCOCH_2_-5, Me-18, Me-19, Me-28, and the furan-3-yl group at C-17 are α-oriented, and 3-tigloyloxy group, Me-29, and H-30 are β-oriented. In addition, a strong NOESY interaction between H-15 and H-30 supported the α-configuration of the epoxy ring. Compound **1** showed a similar CD Cotton effect at 291 nm as the analogous limonoid, humilinolide F (**2**) [15]. Thus, both possessed the same stereo-configuration. On the basis of the evidence above, swietemacrophin was elucidated as structure **1**. This structure was supported by ^1^H-^1^H COSY and NOESY (Table 1) experiments, and ^13^C-NMR assignments were confirmed by DEPT, HSQC, and HMBC techniques (Table 2).

### 2.3. Structure Identification of the Known Isolates

The known isolates were readily identified by comparison of their physical and spectroscopic data (UV, IR, ^1^H-NMR, [α]_D_, and MS) with those of the corresponding authentic samples or literature values. They include five limonoids: humilinolide F (**2**) [15], 3,6-*O*,*O*-diacetylswietenolide (**3**) [16], 3-*O*-tigloylswietenolide (**4**) [16], swietemahonin E (**5**) [17], and swietenine (**6**) [16]. Compound **2** was isolated from *S. macrophylla* for the first time.

**Table 1:** ^1^H-NMR, NOESY, and HMBC data of **1** and **2**.

Atom	1 ^a^			2 ^a,b^
	δ_H_	NOESY	HMBC	δ_H_
H-3	5.71 s	28, 2-AcO	1, 2, 5, 30, 1′	5.72 s
H-5	3.23 dd (*J* = 9.0, 2.5)	6, 29	1, 3, 4, 7	3.45 s
H-6	2.33–2.37 m	19, 28	4, 7, 10	5.49 s
H-9	1.92–1.96 m	11, 19	1, 12, 14	1.95 m
H-11	1.79 m	12	13	
	1.90 m	12	8, 13	
H-12	1.44 m	11	9	
	1.99 m	11	9, 17	
H-14	1.61 m	15	16	
H-15	2.81 dd (*J* = 17.0, 6.5)	14, 30	8, 13, 16	2.80 dd (*J* = 17.0, 6.0)
	3.43 dd (*J* = 17.0, 12.5)	30	8, 16	3.42 dd (*J* = 16.0, 1.3)
H-17	5.18 s	12, 21, 22	12, 14, 16, 21	5.18 s
H-18	1.01 s	12, 22	12, 14, 17	1.01 s
H-19	1.17 s	6, 9	1, 5, 10	1.29 s
H-21	7.49 br s	17	17, 20, 22	7.46 dd (*J* = 1.8, 0.9)
H-22	6.44 br s	17, 18	17, 21	6.41 dd (*J* = 1.8, 0.9)
H-23	7.43 br s	22	20, 21	7.43 dd (*J* = 1.8, 1.8)
H-28	0.79 s	3, 6	3, 4, 5	1.18 s
H-29	0.81 s	5, 3′	3, 5, 28	0.92 s
H-30	3.49 s	15	1, 2, 9	3.48 s
H-3′	7.04 br q (*J* = 7.0)	29, 4′	1′, 2′, 5′	6.98 qq (*J* = 7.5, 1.8)
H-4′	1.93 br *d* (*J* = 7.0)	3′, 5′	2′, 3′	1.94 d (*J* = 7.5)
H-5′	1.97 s	4′	1′, 2′, 3′	1.93 s
2-OAc	2.18 s	3	2-O*C*OMe	2.18 s
6-OAc				2.19 s
7-OMe	3.74 s	6	7	3.82 s

^a^ Recorded in CDCl_3_ at 500 MHz. Values in ppm (δ). *J* (in Hz) in parentheses. ^b^ **2** = humilinolide F [15].

**Table 2:** ^13^C-NMR data of **1** and **2**.

Position	1 ^a,b^	2 ^a,c^
1	206.1	206.0
2	80.7	80.8
3	85.6	85.6
4	40.2	42.1
5	42.4	45.1
6	33.2	72.0
7	174.2	171.1
8	62.7	62.6
9	55.3	55.3
10	50.5	50.6
11	19.7	19.9
12	33.2	33.4
13	36.2	36.1
14	45.0	44.9
15	33.3	33.1
16	169.3	169.2
17	79.3	79.5
18	26.5	26.7
19	16.1	16.1
20	120.3	120.2
21	141.0	140.9
22	110.2	110.1
23	143.3	143.3
28	22.0	21.0
29	20.5	21.3
30	65.3	65.2
1′	166.8	166.6
2′	127.6	127.4
3′	139.7	139.7
4′	14.8	14.9
5′	12.6	12.6
2-OCO*Me*	22.5	22.5
2-O*C*OMe	171.0	171.1
6-OCO*Me*		23.9
6-O*C*OMe		169.7
7-OMe	52.4	53.3

^a^ Recorded in CDCl_3_ at 125 MHz; δ in ppm. Values in ppm (δ); ^b^ Assignments were established from HMQC, HMBC, and DEPT spectra; ^c^ **2** = humilinolide F [15].

### 2.4. Biological Studies

Reactive oxygen species (ROS) [e.g., superoxide anion (O_2_^•−^), hydrogen peroxide] and granule proteases (e.g., elastase, cathepsin G) produced by human neutrophils contribute to the pathogenesis of inflammatory diseases. Inhibition of neutrophil O_2_^•−^ generation by drugs has been proposed as a way to ameliorate inflammatory diseases. The anti-inflammatory effects of the isolated compounds from the seed of *S. macrophylla* were evaluated by suppressing fMet-Leu-Phe (fMLP)-induced O_2_^•−^ generation by human neutrophils. The anti-inflammatory activity data are shown in Table 3. LY294002, a phosphatidylinositol-3-kinase inhibitior, was used as a positive control for superoxide anion generation. From the results of our biological tests, the following conclusions can be drawn: (a) Swietemacrophin (**1**), humilinolide F (**2**), 3,6-*O*,*O*-diacetylswietenolide (**3**), 3-*O*-tigloylswietenolide (**4**), and swietemahonin E (**5**) exhibited inhibition (IC_50_ ≤ 45.44 μM) of superoxide anion generation by human neutrophils in response to formyl-l-methionyl-l-leucyl-l-phenylalanine (fMLP). (b) Among the limonoid analogues **1**–**6**, compounds **1**, **2**, and **5**, with the 8α,30α-epoxy group, and compounds **3** and **4**, with the double bond between C-8 and C-14, exhibited more effective inhibition than analogue **6**, with the double bond between C-8 and C-30, against fMLP-induced O_2_^•−^ generation. (c) Humilinolide F (**2**) is the most effective among the isolated compounds, with IC_50_ = 27.13 ± 1.82 μM, against fMLP-induced superoxide anion generation.

**Table 3:** Inhibitory effects of compounds **1**–**6** from the seed of *S. macrophylla* on superoxide radical anion generation by human neutrophils in response to fMet-Leu-Phe.

Compounds	IC_50_ (μM) ^a^
Swietemacrophin (**1**)	45.44 ± 3.76 *
Humilinolide F (**2**)	27.13 ± 1.82 **
3,6-*O*,*O*-Diacetylswietenolide (**3**)	29.36 ± 1.75 *
3-*O*-Tigloylswietenolide (**4**)	35.58 ± 2.12
Swietemahonin E (**5**)	33.64 ± 2.05 *
Swietenine (**6**)	>100
LY294002 ^b^	1.12 ± 0.11 *

^a^ The IC_50_ values were calculated from the slope of the dose-response curves (SigmaPlot). Values are expressed as average ± SEM (*n* = 4). * *p* < 0.05, ** *p* < 0.01 compared with the control value (DMSO); ^b^ LY294002, a phosphatidylinositol-3-kinase inhibitior, was used as a positive control for superoxide anion generation.

Nitric oxide (NO) is a mediator in the inflammatory response involved in host defense. The anti-inflammatory effects of the compounds isolated from the seed of *S. macrophylla* were also evaluated by suppressing lipopolysaccharide (LPS)-induced NO generation in murine macrophage. The inhibitory activity data of the isolated compounds **1**–**6** against LPS-induced NO generation by macrophages are compiled in Table 4. Quercetin which was reported to inhibit NO production by LPS-stimulated macrophage [18,19] was used as the positive control. From the results of our anti-inflammatory tests, the following conclusions could be drawn: (a) Swietemacrophin (**1**), 3-*O*-tigloylswietenolide (**4**), swietemahonin E (**5**), and swietenine (**6**) exhibited inhibition (IC_50_ ≤ 36.32 μM) of NO generation by murine macrophages in response to LPS. (b) Among the limonoid analogues **1**–**6**, compound **1**, without any substituted group at C-6, and compounds **4**–**6**, with the 6-hydroxy group, exhibited more effective inhibition than their analogues **2** and **3**, with the 6-acetoxy group, against LPS-induced NO generation. (c) Swietemahonin E (**5**) is the most effective among the isolated compounds, with IC_50_ = 29.70 ± 2.11 μM, against LPS-induced NO generation. (d) Cytotoxic effects were determined using the MTT assay. The high cell viability (>92%) indicated that the inhibitory activities of compounds **1**, **4**, **5**, and **6** against LPS-induced NO production did not resulted from their cytotoxicities.

**Table 4:** Inhibitory effects of compounds **1**–**6** from the seed of *S. macrophylla* on nitric oxide (NO) generation by RAW264.7 murine macrophages in response to lipopolysaccharide (LPS).

Compounds	IC_50_ (μM) ^a^
Swietemacrophin (**1**)	33.45 ± 1.88 **
Humilinolide F (**2**)	49.36 ± 4.01
3,6-*O*,*O*-Diacetylswietenolide (**3**)	64.21 ± 5.67
3-*O*-Tigloylswietenolide (**4**)	32.62 ± 3.27 **
Swietemahonin E (**5**)	29.70 ± 2.11 *
Swietenine (**6**)	36.32 ± 2.84
Quercetin ^b^	32.24 ± 2.05 *

^a^ The IC_50_ values were calculated from the slope of the dose-response curves (SigmaPlot). Values are expressed as average ± SEM (*n* = 4). * *p* < 0.05, ** *p* < 0.01 compared with the control; ^b^ Quercetin was used as a positive control.

### 2.5. Discussion

A new limonoid, swietemacrophin (**1**), and five known compounds **2**–**6** were isolated from the seeds of *S. macrophylla*. The structure of new compound **1** was determined by NMR and MS analyses. Among the known isolates, compound **2** has been found for the first time in this plant species. The discovery of more new compounds from the genus *Swietenia* may not only provide more structure-activity data of the isolates, but may also contribute to enhancing our understanding of the taxonomy and evolution of the genus *Swietenia*.

Human neutrophils are known to play a significant role in the host defense against microorganisms and in the pathogenesis of various diseases such as asthma, rheumatoid arthritis, ischemia-reperfusion injury, and chronic obstructive pulmonary disease [20,21]. In response to different stimuli, activated neutrophils secrete a series of cytotoxins, such as superoxide anion (O_2_^•−^), a precursor of other reactive oxygen species (ROS), and bioactive lipids [20,22,23]. Suppression of the extensive or inappropriate activation of neutrophils by drugs has been proposed as a way to ameliorate inflammatory diseases. Based on the results of our biological tests (Table 3), humilinolide F (**2**), 3,6-*O*,*O*-diacetylswietenolide (**3**), and 3-*O*-tigloylswietenolide (**4**) exhibited potent inhibition with IC_50_ values of 27.13 ± 1.82, 29.36 ± 1.75, and 35.58 ± 2.12 μM, respectively, against fMLP-induced superoxide anion generation.

NO is a physiological and a pathological mediator thought to be involved in inflammation [9]. Swietemacrophin (**1**), 3-*O*-tigloylswietenolide (**4**), swietemahonin E (**5**), and swietenine (**6**) showed potent inhibition with IC_50_ values of 33.45 ± 1.88, 32.62 ± 3.27, 29.70 ± 2.11, and 36.32 ± 2.84 μM, respectively, against LPS-induced NO generation.

The above findings indicated that the promising inhibitory activity against fMLP-induced O_2_^•−^ generation and LPS-induced NO release of *S. macrophylla* and its isolates could stimulate future development of new anti-inflammatory agents.

## 3. Experimental Section

### 3.1. Ethics Statement

Blood was taken from healthy human donors (20–30 years old) by venipuncture, using a protocol approved by the Institutional Review Board at Chang Gung Memorial Hospital. All donors gave written consent. The Medical Ethics Committee of Chang Gung Memorial Hospital approved this consent procedure.

### 3.2. General Experimental Procedures

Melting points were determined on a Yanaco micro-melting point apparatus (Yanaco, Kyoto, Japan) and were uncorrected. Optical rotations were measured using a Jasco DIP-370 polarimeter (Jasco Co., Hachioji, Japan) in CHCl_3_. Ultraviolet (UV) spectra were obtained on a Jasco UV-240 spectrophotometer (Jasco Co.). Circular dichroism (CD) spectra were recorded on a Jasco J-810 spectropolarimeter (Jasco Co.). Infrared (IR) spectra (neat or KBr) were recorded on a Perkin Elmer 2000 FT-IR spectrometer (Perkin-Elmer Corp., Waltham, MA, USA). Nuclear magnetic resonance (NMR) spectra, including correlation spectroscopy (COSY), nuclear Overhauser effect spectrometry (NOESY), heteronuclear multiple-bond correlation (HMBC), and heteronuclear single-quantum coherence (HSQC) experiments, were acquired using a Varian Unity 400 or a Varian Inova 500 spectrometer (Varian, Palo Alto, CA, USA) operating at 400 and 500 MHz (^1^H) and 100 and 125 MHz (^13^C), respectively, with chemical shifts given in ppm (δ) using tetramethylsilane (TMS) as an internal standard. Electrospray ionisation (ESI) and high-resolution electrospray ionization (HRESI)-mass spectra were recorded on a Bruker APEX II (Bruker, Billerica, MA, USA) or a VG Platform Electrospray ESI/MS mass spectrometer (VG Biotech, Altrincham, UK). Silica gel (70–230, 230–400 mesh, Merck, Darmstadt, Germany) was used for column chromatography (CC). Silica gel 60 F-254 (Merck) was used for thin-layer chromatography (TLC) and preparative thin-layer chromatography (PTLC).

### 3.3. Plant Material

The seeds of *S. macrophylla* were collected from Tajen University, Pintung County, Taiwan, in February 2010 and identified by S.-Z. Yang (Department of Forest Resources, Management and Technology, National Pingtung University of Science and Technology, Pingtung, Taiwan). A voucher specimen (No. 63699) was deposited at the herbarium of the Department of Forest Resources, Management and Technology, National Pingtung University of Science and Technology.

### 3.4. Extraction and Isolation

The dried seeds (380 g) of *S. macrophylla* were pulverized and extracted with MeOH (3 × 2 L) at room temperature for 3 days. The extract was concentrated under reduced pressure at 35 °C, and the residue (34.5 g) was partitioned between EtOAc and H_2_O (1:1) to provide the EtOAc-soluble fraction (Fr. A; 12.4 g). The H_2_O-soluble fraction was further extracted with *n*-BuOH, and the *n*-BuOH-soluble part (Fr. B; 9.3 g) and the H_2_O-soluble one (Fr. C; 12.7 g) were separated. Fraction A (12.4 g) was purified by CC (SiO_2_ (565 g), 70–230 mesh; CH_2_Cl_2_/MeOH gradient) to afford 10 fractions: Fr. A1 (eluted with 1.0 L of CH_2_Cl_2_), Fr. A2 (800 mL, CH_2_Cl_2_/MeOH 95:1), Fr. A3 (850 mL, CH_2_Cl_2_/MeOH 90:1), Fr. A4 (800 mL, CH_2_Cl_2_/MeOH 80:1), Fr. A5 (1.2 L, CH_2_Cl_2_/MeOH 50:1), Fr. A6 (1.0 L, CH_2_Cl_2_/MeOH 30:1), Fr. A7 (800 mL, CH_2_Cl_2_/MeOH 10:1), Fr. A8 (900 mL, CH_2_Cl_2_/MeOH 5:1), Fr. A9 (850 mL, CH_2_Cl_2_/MeOH 1:1), and Fr. A10 (1.5 L, MeOH). Fraction A3 (1.15 g) was purified by CC (SiO_2_ (52 g), 230–400 mesh; CH_2_Cl_2_/acetone 10:1 to 1:1, 450-mL fractions) to give nine subfractions: Frs. A3-1–A3-9. Fraction A3-3 (125 mg) was further purified by preparative TLC (SiO_2_; CH_2_Cl_2_/acetone 20:1) to yield **1** (4.6 mg). Fraction A3-4 (117 mg) was further purified by preparative TLC (SiO_2_; CH_2_Cl_2_/EtOAc 15:1) to yield **2** (4.2 mg). Fraction A3-5 (138 mg) was further purified by preparative TLC (SiO_2_; CHCl_3_/acetone 15:1) to yield **3** (5.5 mg). Fraction A3-6 (122 mg) was further purified by preparative TLC (SiO_2_; CH_2_Cl_2_/acetone 10:1) to afford **4** (4.4 mg). Fraction A4 (1.23 g) was subjected to CC (SiO_2_ (56 g), 230–400 mesh; CH_2_Cl_2_/MeOH 12:1 to 0:1, 300-mL fractions) to afford ten subfractions: Frs. A4-1–A4-10. Fraction A4-3 (105 mg) was further purified by preparative TLC (SiO_2_; CH_2_Cl_2_/acetone 7:1) to give **5** (5.2 mg). Fraction A5 (1.07 g) was subjected to CC (SiO_2_ (50 g), 230–400 mesh; CHCl_3_/MeOH 9:1 to 0:1, 350-mL fractions) to afford eight subfractions: Frs. A5-1–A5-8. Fraction A5-4 (128 mg) was further purified by preparative TLC (SiO_2_; CH_2_Cl_2_/MeOH 7:1) to afford **6** (19.5 mg).

*Swietemacrophin* (**1**). Colorless needles (CH_2_Cl_2_/MeOH), m.p. 114–116 °C. ${\left[\text{α}\right]}_{D}^{25}$: *–*27.2 (*c* 0.16, CHCl_3_). UV (MeOH): λ_max_ (log ε) = 215 (4.08) nm. CD (MeOH, Δε): 291 (*–*13.96 × 10^3^) nm. IR (KBr): υ_max_ = 1735 (C=O), 1721 (C=O), 1708 (C=O) cm^−1^. ^1^H-NMR spectroscopic data, see Table 1. ^13^C-NMR spectroscopic data, see Table 2. ESI-MS: *m*/*z* = 649 [M + Na]^+^. HR-ESI-MS: *m*/*z* = 649.2622 [M + Na]^+^ (calcd for C_34_H_42_O_11_Na: 649.2625).

*Humilinolide F* (**2**). Colorless needles (EtOAc); m.p. 113*–*115 °C. ${\left[\text{α}\right]}_{\text{D}}^{25}$: *–*42.8 (*c* 0.15, CHCl_3_). IR (KBr): υ_max_ 1755 (C=O), 1743 (C=O), 1711 (C=O) cm^−1^. ^1^H-NMR (CDCl_3_, 500 MHz): δ = 0.92 (3H, s, H-29), 1.01 (3H, s, H-18), 1.18 (3H, s, H-28), 1.29 (3H, s, H-19), 1.93 (3H, s, H-5′), 1.94 (3H, d, *J* = 7.5 Hz, H-4′), 1.95 (1H, m, H-9), 2.18 (3H, s, OAc-2), 2.19 (3H, s, OAc-6), 2.81 (1H, dd, *J* = 17.0, 6.0 Hz, H-15), 3.42 (1H, dd, *J* = 17.0, 1.5 Hz, H-15), 3.45 (1H, s, H-5), 3.48 (1H, s, H-30), 3.82 (3H, s. OMe-7), 5.19 (1H, s, H-17), 5.50 (1H, s, H-6), 5.72 (1H, s, H-3), 6.41 (1H, br s, H-22), 6.98 (1H, br q, *J* = 7.5 Hz, H-3′), 7.43 (1H, br s, H-23), 7.47 (1H, br s, H-21). ESI-MS: *m*/*z* = 329 [M + Na]^+^.

*3,6-O,O-Diacetylswietenolide* (**3**). Colorless needles (EtOAc); m.p. 155*–*157 °C. ${\left[\text{α}\right]}_{\text{D}}^{25}$: *–*15.4 (*c* 0.18, CHCl_3_). IR (KBr): υ_max_ = 1745 (C=O), 1727 (C=O) cm^−1^. ^1^H-NMR (CDCl_3_, 500 MHz): δ = 0.86 (3H, s, H-29), 1.05 (3H, s, H-18), 1.08 (3H, s, H-28), 1.17 (3H, s, H-19), 1.18 (1H, m, H-11), 1.78 (1H, m, H-12), 1.83 (1H, m, H-11), 1.90 (1H, m, H-12), 2.10 (1H, m, H-9), 2.15 (1H, dd, *J* = 15.0, 5.5 Hz, H-30), 2.16 (3H, s, OAc-3), 2.18 (3H, s, OAc-6), 2.81 (1H, dd, *J* = 15.0, 2.0 Hz, H-30), 3.16 (1H, ddd, *J* = 10.0, 5.5, 2.0 Hz, H-2), 3.41 (1H, br s, H-5), 3.45 (1H, br d, *J* = 16.0 Hz, H-15), 3.69 (1H, d, *J* = 16.0 Hz, H-15), 3.76 (3H, s. OMe-7), 4.88 (1H, d, *J* = 10.0 Hz, H-3), 5.47 (1H, br s, H-6), 5.61 (1H, s, H-17), 6.47 (1H, br s, H-22), 7.43 (1H, br s, H-23), 7.54 (1H, br s, H-21). ESI-MS: *m*/*z* = 593 [M + Na]^+^.

*3-O-Tigloylswietenolide* (**4**). Colorless needles (*n*-hexane-EtOAc); m.p. 209*–*210 °C. ${\left[\text{α}\right]}_{\text{D}}^{25}$: *–*13.8 (*c* 0.16, CHCl_3_). IR (KBr): υ_max_ = 3486 (OH), 1735 (C=O), 1712 (C=O) cm^−1^. ^1^H-NMR (CDCl_3_, 500 MHz): δ = 0.86 (3H, s, H-29), 1.00 (3H, s, H-18), 1.10 (3H, s, H-28), 1.17 (1H, m, H-12), 1.43 (3H, s, H-19), 1.74 (1H, m, H-12), 1.76 (1H, m, H-11), 1.83 (3H, d, *J* = 7.0 Hz, Me-3′), 1.88 (1H, m, H-11), 1.88 (3H, s, Me-2′), 2.11 (1H, m, H-9), 2.12 (1H, m, H-30), 2.66 (1H, dd, *J* = 15.5, 2.5 Hz, H-30), 2.82 (1H, s, D_2_O exchangeable, OH-6), 3.21 (1H,dd, *J* = 9.5, 6.0 Hz, H-2), 3.25 (1H, dt, *J* = 20.5, 3.0 Hz, H-15), 3.38 (1H, br s, H-5), 3.54 (1H, dt, *J* = 20.5, 1.5 Hz, H-15), 3.86 (3H, s, OMe-7), 4.56 (1H, br s, H-6), 4.71 (1H, d, *J* = 9.5 Hz, H-3), 5.43 (1H, s, H-17), 6.40 (1H, br s, H-22), 6.93 (1H, q, *J* = 7.0 Hz, H-3′), 7.43 (1H, br s, H-23), 7.48 (1H, br s, H-21). ESI-MS: *m*/*z* = 591 [M + Na]^+^.

*Swietemahonin E* (**5**). Colorless needles (EtOAc); m.p. 151*–*153 °C. ${\left[\text{α}\right]}_{\text{D}}^{25}$: *–*19.6 (*c* 0.15, CHCl_3_). IR (KBr): υ_max_ = 3492 (OH), 1733 (C=O), 1715 (C=O) cm^−1^. ^1^H-NMR (CDCl_3_, 500 MHz): δ = 0.93 (3H, s, H-29), 1.04 (3H, s, H-18), 1.12 (3H, s, H-28), 1.35 (3H, s, H-19), 1.38 (1H, m, H-12), 1.54 (1H, dd, *J* = 12.0, 7.5 Hz, H-14), 1.77 (1H, m, H-11), 1.92 (1H, m, H-11), 1.93 (3H, d, *J* = 7.0 Hz, Me-3′), 1.94 (3H, s, Me-2′), 2.10 (1H, m, H-12), 2.01 (1H, dd, *J* = 14.0, 4.5 Hz, H-9), 2.71 (1H,dd, *J* = 17.5, 7.5 Hz, H-15), 2.92 (1H, br s, D_2_O exchangeable, OH-6), 3.10 (1H, d, *J* = 2.5 Hz, H-30), 3.16 (1H, dd, *J* = 17.5, 12.0 Hz, H-15), 3.36 (1H, s, H-5), 3.62 (1H, dd, *J* = 9.5, 2.5 Hz, H-2), 3.96 (3H, s. OMe-7), 4.46 (1H, s, H-6), 4.85 (1H, d, *J* = 9.5 Hz, H-3), 5.08 (1H, s, H-17), 6.34 (1H, br s, H-22), 7.02 (1H, q, *J* = 7.0 Hz, H-3′), 7.40 (1H, br s, H-21), 7.43 (1H, t, *J* = 2.0 Hz, H-23). ESI-MS: *m*/*z* = 607 [M + Na]^+^.

*Swietenine* (**6**). Colorless needles (MeOH); m.p. 275–277 °C. ${\left[\text{α}\right]}_{\text{D}}^{25}$: *–*18.5 (*c* 0.15, CHCl_3_). IR (KBr): υ_max_ = 3492 (OH), 1733 (C=O), 1715 (C=O) cm^−1^. ^1^H-NMR (CDCl_3_, 500 MHz): δ = 0.89 (3H, s, H-29), 0.97 (3H, s, H-18), 1.12 (3H, s, H-28), 1.45 (3H, s, H-19), 1.46 (1H, ddd, *J* = 17.0, 11.0, 4.0 Hz, H-12), 1.74 (3H, d, *J* = 7.0 Hz, Me-3′), 1.77 (1H, m, H-12), 1.81 (1H, m, H-11), 1.81 (3H, s, Me-2′), 2.05 (1H, qd, *J* = 13.0, 4.0 Hz, H-11), 2.23 (1H,dd, *J* = 6.0, 1.5 Hz, H-14), 2.30 (1H,dd, *J* = 13.0, 4.0 Hz, H-9), 2.76 (1H, dd, *J* = 19.0, 1.5 Hz, H-15), 2.82 (1H,dd, *J* = 19.0, 6.0 Hz, H-15), 2.89 (1H, s, D_2_O exchangeable, OH-6), 3.50 (1H, s, H-5), 3.52 (1H, dd, *J* = 9.5, 7.5 Hz, H-2), 3.76 (3H, s. OMe-7), 4.56 (1H, s, H-6), 4.64 (1H, d, *J* = 9.5 Hz, H-3), 5.30 (1H, d, *J* = 7.5 Hz, H-30), 5.55 (1H, br s, H-17), 6.39 (1H, br s, H-22), 4.87 (1H, q, *J* = 7.0 Hz, H-3′), 7.45 (1H, t, *J* = 1.5 Hz, H-23), 7.56 (1H, br s, H-21). ESI-MS: *m*/*z* = 591 [M + Na]^+^.

### 3.5. Biological Assay

The effect of the isolated compounds on neutrophil pro-inflammatory response was evaluated by monitoring the inhibition of superoxide anion generation in fMLP-activated human neutrophils in a concentration-dependent manner. The purity of the tested compounds was >98% as identified by NMR and MS.

#### 3.5.1. Preparation of Human Neutrophils

Human neutrophils from venous blood of healthy, adult volunteers (20–30 years old) were isolated using a standard method of dextran sedimentation prior to centrifugation in a Ficoll Hypaque gradient and hypotonic lysis of erythrocytes [24]. Purified neutrophils containing >98% viable cells, as determined by the trypan blue exclusion method [25], were resuspended in a calcium (Ca^2+^)-free HBSS buffer at pH 7.4 and were maintained at 4 °C prior to use.

#### 3.5.2. Measurement of Superoxide Anion Generation

The assay for measurement of O_2_^•−^ generation was based on the SOD-inhibitable reduction of ferricytochrome *c* [26]. In brief, neutrophils (1 × 10^6^ cells/mL) pretreated with the various test agents at 37 °C for 5 min were stimulated with fMLP (1 μmol/L) in the presence of ferryicytochrome *c* (0.5 mg/mL). Extracellular O_2_^•−^ production was assessed with a UV spectrophotometer at 550 nm (Hitachi U-3010, Tokyo, Japan). The percentage of superoxide inhibition of the test compound was calculated as the percentage of inhibition = {(control – resting) – (compound – resting)}/(control – resting) × 100. The software, SigmaPlot was used for determining the IC_50_ values.

#### 3.5.3. Determination of NO Production

The murine macrophage cell line RAW264.7 was cultured in Dulbecco’s modified Eagle’s medium (DMEM, Gibco BRL, Life Technologies Inc., Burlington, ON, Canada) supplemented with 10% heat-inactivated fetal bovine serum (FBS) and incubated at 37 °C in a humidified 5% CO_2_ atmosphere with a 96-well flat-bottomed culture plate. After 24 h, the condition medium was replaced with fresh DMEM; and FBS. Then, compounds **1**–**6** (0, 1, 2.5, 5, 10, and 20 μg/mL) were added, respectively, in the presence of lipopolysaccharide (LPS; 1 μg/mL; Cat No: L-2654, Sigma-Aldrich Co., St. Louis, MO, USA) and incubated under the same condition for 24 h. The cultured cells were then centrifuged, and the supernatants were used for NO-production measurement. The supernatant was mixed with an equal volume of the Griess reagent (1% sulfanilamide, 0.1% *N*-(naphthalen-1-yl)ethylenediamine dihydrochloride in 2.5% H_2_PO_4_ soln.) and incubated for 10 min at room temperature. Nitrite concentration was determined by measuring the absorbance at 540 nm using an ELISA plate reader (Anthos Labtec Instruments, Salzburg, Austria) [27]. The percentage of NO inhibition of the test compound was calculated as follows: inhibitory rate (%) = (1 − (LPS/sample − untreated)/(LPS − untreated) × 100. All tests were run in triplicate and averaged. The data were expressed as a mean of three experiments. The software SigmaPlot was used for determining the IC_50_ values.

#### 3.5.4. Cell Viability Assay

A MTT colorimetric assay was used to determine cell viability. The assay was modified from that of Mosmann [28]. The test is based upon the selective ability of living cells to reduce the yellow soluble salt, MTT, to a purple-blue insoluble formazan. MTT (Merck; dissolved in phosphate-buffered saline at 5 mg/mL) soln. was added onto the attached cells mentioned above (10 μL per 100 μL culture) and incubated at 37 °C for 4 h. Then, DMSO was added, and amount of colored formazan metabolite formed was determined by absorbance at 550 nm. The optical density of formazan formed in control (untreated) cells was taken as 100% viability.

#### 3.5.5. Statistical Analysis

Results are expressed as the mean ± SEM, and comparisons were made using Student’s *t*-test. A probability of 0.05 or less was considered significant. The software SigmaPlot was used for the statistical analysis.

## 4. Conclusions

Six compounds, including a new compound **1**, were isolated from the seed of *S. macrophylla*. The structures of these compounds were established on the basis of spectroscopic data. Reactive oxygen species (ROS) [e.g., superoxide anion (O_2_^•−^), hydrogen peroxide] produced by human neutrophils contribute to the pathogenesis of inflammatory diseases. The effects on neutrophil pro-inflammatory responses of isolates were evaluated by suppressing fMLP-induced O_2_^•−^ generation by human neutrophils. The results of anti-inflammatory experiments indicate that compounds **1**–**5** can significantly inhibit fMLP-induced O_2_^•−^ generation. Humilinolide F (**2**), 3,6-*O*,*O*-diacetylswietenolide (**3**), and 3-*O*-tigloylswietenolide (**4**) are the most effective among the isolated compounds, with IC_50_ values of 27.13 ± 1.82, 29.36 ± 1.75, and 5.58 ± 2.12 μM, respectively, against fMLP-induced superoxide anion generation. Furthermore, compounds **1**, **4**, **5**, and **6** showed potent inhibition with IC_50_ values of 33.45 ± 1.88, 32.62 ± 3.27, 29.70 ± 2.11, and 36.32 ± 2.84 μM, respectively, against LPS-induced NO generation. Thus, our study suggests *S. macrophylla* and its isolates could be further developed as potential candidates for the treatment or prevention of various inflammatory diseases.
